# Two-Layered Graph-Cuts-Based Classification of LiDAR Data in Urban Areas

**DOI:** 10.3390/s19214685

**Published:** 2019-10-28

**Authors:** Yetao Yang, Ke Wu, Yi Wang, Tao Chen, Xiang Wang

**Affiliations:** 1 Institute of Geophysics and Geomatics, China University of Geosciences, Wuhan 430074, China; 2 State Key Laboratory of Bridge Structure Health and Safety, Wuhan 430034, China

**Keywords:** LiDAR point cloud, classification, graph cuts, hierarchical graph

## Abstract

Classifying the LiDAR (Light Detection and Ranging) point cloud in the urban environment is a challenging task. Due to the complicated structures of urban objects, it is difficult to find suitable features and classifiers to efficiently category the points. A two-layered graph-cuts-based classification framework is addressed in this study. The hierarchical framework includes a bottom layer that defines the features and classifies point clouds at the point level as well as a top layer that defines the features and classifies the point cloud at the object level. A novel adaptive local modification method is employed to model the interactions between these two layers. The iterative graph cuts algorithm runs around the bottom and top layers to optimize the classification. In this way, the addressed framework benefits from the integration of point features and object features to improve the classification. The experiments demonstrate that the proposed method is capable of producing classification results with high accuracy and efficiency.

## 1. Introduction

Three dimensional (3D) geo-spatial information is important in a variety of urban applications such as urban planning, disaster simulation, environment studies, and ecology assessments. LiDAR (Light Detection and Ranging) is particularly useful for the 3D representation of urban areas due to the availability of dense point clouds [1]. LiDAR data processing tasks, such as point clouds classification and 3D urban objects acquisition, however, are very challenging [2]. There are different types of objects, such as buildings, trees, low vegetation, and cars, which can be found in a small neighborhood environment. Thus, these objects have a great chance of exhibiting overlapping or even mixing in 3D space. At the same time, urban objects, especially manufactured objects, show a wide variation in appearance, which confuses the definition of them. The complexity of the urban environment makes it difficult to acquire accurate 3D information from LiDAR point clouds.

When addressing the task of 3D point cloud classification, there are typically two kinds of approaches mentioned in current literature: point-based approaches and object-based approaches. The point-based approaches use supervised statistical or machine learning classification methods running on discrete features generated from individual points with neighbors [3,4,5,6,7], while the object-based approaches first segment points into objects, and then the classification process is performed on the object-based features [8,9,10,11,12,13]. Both the point-based approaches and the object-based approaches have advantages related to certain aspects of point cloud classification [14]. However, these two kinds of approaches also have obvious drawbacks for point cloud classification in the urban environment. For point-based approaches, a constant neighborhood parameter (i.e., either a fixed radius or a constant nearest k) across all 3D points is selected to design features with respect to heuristic or empiric knowledge. The designed features with definite neighborhoods cannot represent the complex structures in urban scenes. Regarding the object-based approaches, it is not easy to evaluate the parameters for segmentation [15]. The complex structures of the urban scene tend to result in mistakes in segmentation, such as the formation of spurious objects (segments that do not exist in reality), over-segmented objects (one actual object is segmented into multiple segments), and under-segmented objects (multiple objects are segmented into one segment). Poor segmentation will affect the classification accuracy [15,16].

In order to avoid strong assumptions on designed features for the point-based approaches, recent works have introduced deep learning methods to acquire features [17,18,19]. This kind of method is capable of automatically learning deep features from the training data, which helps to overcome defects such as hand-crafted features and definite neighborhood parameters. The features from deep learning methods show the ability to represent high-level semantic structures [20]. However, deep learning technology is deficient for handling unordered data from point clouds. At the same time, it needs mass training samples and introduces high computational effort that tends to be impracticable for large-scale urban scenes. Besides deep learning methods, another simple method is to calculate an optimal neighborhood size for each individual 3D point [21,22]. This method also requires high computational effort. Integrating the contexture information into the point-based approaches is also a recent trend that can achieve improvements. For example, some point-based methods incorporate contexture information by adopting Markov Random Field (MRF) [23,24] or Conditional Random Fields (CRF) [7,25]. However, the contexture information only involves a small neighborhood and considers only point-level interactions [26].

According to the limitations of point-based and object-based classifiers, some works try to combine them to establish hybrid approaches. Kim and Sohn proposed a method using multiple classifiers (Random Forest) to integrate point-based and object-based classification results [27]. In this study, the line and plane segments were obtained from the random sample consensus (RANSAC) and the minimum description length (MDL) and then evaluated with spatial constraints. Point-based features and object-based features were extracted and input for two Random Forest classifiers separately. Xu et al. proposed a multiple-entity-based classification system using features from three different entities: points, plane segments, and line segments [28]. The features extracted from these entities were input into a four-step classification strategy. Wang et al. proposed a method that integrates point features and object features in a deep learning classification [20]. They use predefined multilevel point clusters to capture the features of objects from coarse to fine. Besides the point-based feature descriptors of all points, cluster-based (object-based) features obtained from max-pooling are input for point cloud classification. In these hybrid methods, the point-based features and object-based features are separately extracted and parallel input into the hybrid classifiers. The defects mentioned above, such as the determination of the neighborhood of individual points or the segmentation of the objects, remain unsettled.

A major challenge of a hybrid approach is the efficient combination of point-based and object-based classification to achieve accurate classification of the urban objects. Actually, point-based classification and object-based classification can provide feedback for one another, which can be used as a constraint to improve the point-based neighborhood terms and the object-based segmentations. Iterative optimization based on energy minimization is a practical way to build the interaction between the two parts of a hybrid approach. In remote sensing image classification, the graph cuts technique is usually used to resolve an energy minimization problem, like Markov Random Fields (MRF). The graph cuts technique constructs a graph for the energy function such that the minimum cut on the graph corresponds to the minimum energy [29,30]. For multi-class applications, the extended algorithms, such as the α-expansion algorithm [31], have been adopted to minimize the energy function.

To overcome these problems, this study introduces a novel two-layered graph-cuts-based framework for point cloud classification in complex urban areas. The main idea of the proposed framework is to design a two-layered hierarchical graph model, where the top layer and the bottom layer represent the spatial structures of segmented objects and original points, respectively. It intends to iteratively optimize the point-based neighborhood and the segmentation of the objects, thus improving the classification accuracy. The main contributions of this paper are as follows:

- A two-layered graph model is developed to integrate the point-based classification and the object-based classification. The bottom layer uses a graph to structure the original points with the support of neighborhood information, and a graph cuts algorithm is performed on this layer to optimize the point-based classification. The top layer builds a graph based on the segmented objects, and the graph cuts algorithm is also adopted to classify the segmented objects. Iterative graph cuts processes around the top layer and bottom layer graph are then performed to improve the model’s accuracy.
- A novel adaptive local modification method is introduced for optimizing the two-layered graph structure during the iteration processes. This method provides bidirectional propagation for the graphs of the top layer and bottom layer; thus, the graphs are efficiently reconstructed to achieve a high classification performance.
- Based on the different types of the objects in the real world, the spatial constraints (object features) are extracted and formulated as the energy terms for the graph cuts algorithm to classify point clouds at the object level. The object features, including height, planarity, and topology, are developed and formulated in the top layer graph.
- The efficiency of the proposed method is shown in comparison with the related methods and other state of art methods. Comparing to the related point classifier and contextual integrated classifier, the overall accuracy increases by 7% and 2.5%, respectively. The proposed method also outperforms most of the state-of-the-art methods on ISPRS benchmark dataset with an overall accuracy of 83.2%.

## 2. Methods

### 2.1. Overview

**Figure 1:**
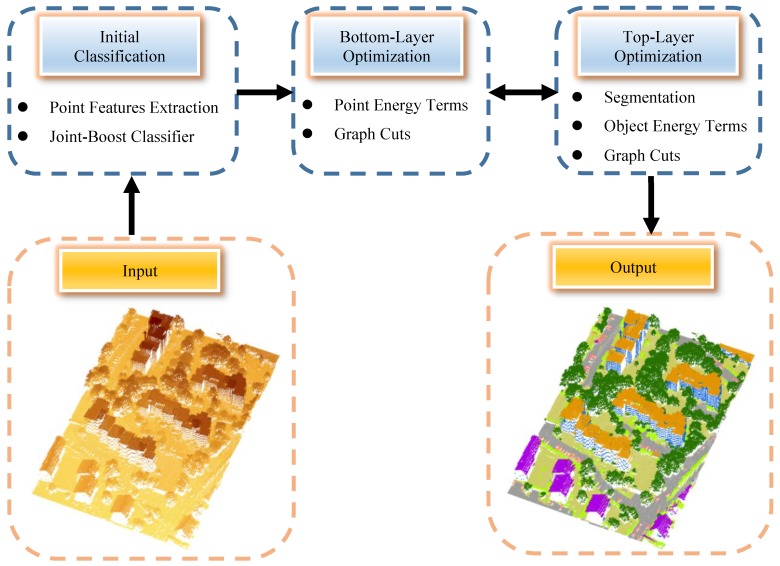
The flow chart of the proposed two-layered graph cuts classification framework.

The overview of the proposed framework is depicted in Figure 1. The whole procedure is composed of three parts: initial classification, bottom layer optimization, and top layer optimization.

(1). An initial classification is performed on individual points by the Joint-Boost algorithm. The point features used for classification are derived from geometry, multi-return, and intensity information.
(2). At the bottom layer, the graph cuts algorithm is used to optimize the classification of points to consider the contextual information. The point energy terms are derived from the initial classification results and the feedback from the top layer.
(3). At the top layer, segmented objects are acquired by clustering the points from the bottom layer according to certain criteria. Then, graph cuts optimization is run on objects to improve the classification. The energy terms of objects are derived from the spatial constraints of nature object classes and the propagation from the bottom layer.

Iterative processes are run around the bottom layer graph and top layer graph to optimize the classification through a novel local adaptive modification. A detailed explanation of the whole procedure as well as the respective methods is presented in the following subsections.

### 2.2. Initial Classification of Point Clouds

The proposed framework employs the graph cuts algorithm for the classification tasks at both the point level and object level. The main idea of the graph cuts algorithm is to effectively cut the edges between the nodes, thus achieving optimal separation of the nodes. A common theme underlying the algorithm is the formation of a weighted graph. For classification purposes, the costs of the nodes and edges in a weighted graph can be derived from the probabilities of the potential labels. Therefore, computing the probabilities of the potential labels for the individual points is the preliminary step for the proposed method. Point-based classifiers with the ability to provide probabilities of the potential labels are ideal tools for initializing the cost of the bottom layer weighted graph. In this study, Joint-Boost was used for the initial labelling task. The Joint-Boost classifier has a remarkable ability to automatically select useful features from a large number of input features [4,32]. In addition, it is able to output probabilities of the potential labels.

#### 2.2.1. Features

Efficient point features are the premise for the LiDAR point cloud classification; therefore, feature selection and calculation are widely discussed in the literature [4,33,34,35]. In this study, features considered to be well-suited to this particular task were adopted for the initial classification of the points, as shown in Table 1. The echo, height, plane, and eigenvalue features were presented by Chehata et al. [33], and the intensity and density features were presented by Weinmann et al. [35]. There are two types of neighborhood, cylinder and sphere, and multi-scale neighborhood sizes are used to extract the features.

The intensity records the reflectance value of the return signals of laser beams. It is related to the incidence angle and distance. If the data are obtained from multiple strips, the intensity needs to be corrected before further process [36]. The number of echoes feature represents the total number of echoes within the waveform of the current LiDAR point, while the ratio of the echoes normalizes the echo number by dividing the total number of the echoes. The height above ground feature is the height value relative to the ground plane extracted by the LAStools lasheight tool [37]. The height variance and curvature can be calculated based on this relative height using a cylinder neighborhood. The curvature feature represents the maximum value of the gradient difference in height, which is computed in four main directions. The variance-covariance matrix is computed within a sphere neighborhood, and the eigenvalues λ_1_ > λ_2_ > λ_3_ are used as features in these studies. Some eigenvalue-derived features, including the omnivariance, planarity, anisotropy, and sphericity, are also included [38]. A local plane derived from a sphere neighborhood is used to compute the normal vector variance and residuals to the local plane feature. The point density feature is the number of points within a given neighborhood divided by its volume. To differentiate vegetation and roofs, sphere and cylinder neighborhoods are both used to compute the point density.

#### 2.2.2. The Joint-Boost Classifier

The Joint-Boost classifier gives a probability P(c=l/Z) for each possible class l∈C based on a feature vector Z, where C is the set of possible labels. The classifier composes a series of simple decision or regression stumps that score each possible class label. A decision or regression stump can be formulated as

(1) $$h\left(Z,l\right)=\left\{ \begin{array}{l} a,\;z^{f}>\theta \;and\;l\in C_{s}\\ b,\;z^{f}<=\theta \;and\;l\in C_{s}\\ c,\;l\notin C_{s} \end{array}\right.$$

where $z^{f}$ denotes the *f*-th feature of the feature vector Z, θ is a threshold, and $C_{s}$ is a subset of possible classes C.

The stumps for a given class label are then added to a strong decision:

(2) $$H(Z,l)=\sum_{l\in C_{s}}h(Z,l).$$

Finally, the probability for a possible class l∈C is given by performing a soft transformation on the strong decision:

(3) $$P(c=l/Z)=\frac{e^{H(Z,l)}}{\sum_{l\in C}e^{H(Z,l)}}.$$

### 2.3. Bottom Layer Graph Formulation

The bottom layer graph is built upon the original point cloud. The nodes in the graph represent the individual data points, while the edges indicate the neighborhood environment. The energy minimization method that intends to smooth the segmentation and enhance the separation (points in a neighborhood are more likely to be assigned to the same class) is employed for multi-class labelling in this study [31,39]. The energy minimization is formulated as

(4) $$E(L_{1})=\overset{data\;\cos t}{\overset{︷}{\sum {}_{p\in P}D_{p}(l_{p})}}+\overset{smooth\;\cos t}{\overset{︷}{\sum {}_{p,q\in N_{P}}w_{pq}\delta (l_{p}\neq l_{q})}}$$

where P represents the set of the points, $N_{P}$ indicates the neighborhood system for points, and δ is an indicator function. The bottom layer graph cuts optimization aims to assign each point p∈P a label $l_{p}\in C$ to minimize the energy $E(L_{1})$.

Two energy terms are considered in this paper. The probabilities of the possible labels acquired from the initial Joint-Boost classification are used to get data cost term in the energy formulation:

(5) $$D_{p}(l_{p})=1-P(c=l/Z).$$

In the bottom layer graph, the smooth cost term uses a sphere system with a definite radius to evaluate the labelling consistency. Since adjacent points are more likely to belong to the same object, the smooth cost has weights that decrease with distance, i.e.,

(6) $$w_{pq}=e^{-{\left(\frac{\left\|p-q\right\|}{d}\right)}^{2}}$$

where d is the average distance between data points [39].

### 2.4. Top Layer Graph Formulation

The optimized results of the bottom layer are used to build the top layer graph. Individual points from the bottom layer are clustered into separated segments that indicate real urban objects. Thus, the segments are formed according to the labelling, norms, and distances of the points. In the top layer graph, the nodes represent the objects, and the edges denote the adjacent relations of these objects. Similarly, the energy minimization method is employed to optimize the top layer graph, which leads to a more accurate classification. However, the formulation of the graph is different in the top layer, as object features are described as energy terms. Since the data points have a probability for each possible class that is acquired by the initial Joint-Boost classification, the average probability can be described by a data energy term $E_{d}(l_{s})$ for each object s∈S as

(7) $$E_{d}(l_{s})=1-\sum_{p\in s}P(c=l/Z)/n$$

where n stands for the number of points in object s.

Some inherent constraints of the real urban objects, such as the height, shape, and spatial relations, can be used to improve the classification performance. In this study, the height of the object h(s) is used to discriminate objects at different height levels. The h(s) of an object is defined as the average height of the individual points within the object. To avoid complex situations and achieve efficient classification of the main urban object types, three height levels, including the top level, middle level, and ground level are defined in this study. Trees and roofs are at the top level, low vegetation and cars are at the middle level, and grass and roads are at the ground level. Based on the elevation feature, a data energy term $E_{h}(l_{s})$ is defined as

(8) $$E_{h}(l_{s})=\left\{ \begin{array}{l} \min(abs(1-\frac{h(s)}{\sigma_{hm}}),1),\;if\;l_{s}\;belongs\;to\;middle\;level\;classes\\ \max(1-\frac{h(s)}{\sigma_{ht}},0),\;if\;l_{s}\;belongs\;to\;top\;level\;classes\\ \min(\frac{h(s)}{\sigma_{hg}},1),\;if\;l_{s}\;belongs\;to\;ground\;level\;classes \end{array}\right.$$

where $\sigma_{hm}$, $\sigma_{ht}$, and $\sigma_{hg}$ are the normalization factors. The normalization factors are empirically determined according to real urban scenes for classification.

Besides the height, the planarity index introduced by Gross and Thoennessen is also employed to improve the classification [38]. Building roofs, roads, and grass have planar surfaces that are different from classes such as trees and low vegetation. The planarity index is important to improve the discrimination of classes with planar surfaces and rough surfaces. It is a supplement to the classes at same height level, such as trees and building roofs. The energy term $E_{g}(l_{s})$ is defined as

(9) $$E_{g}(l_{s})=\left\{ \begin{array}{l} \min(\frac{1-g(s)}{\sigma_{g}},1),\;if\;l_{s}\;belongsto\;classes\;with\;planar\;surface\;\\ 0,\;otherwise \end{array}\right.$$

where $\sigma_{g}$ are the empirically determined normalization factors for planar objects, and g(s) represents the planarity index of object s.

To further classify the planar objects, the direction index k(s) is introduced and defined as the Z-component of the normal vector. The direction index k(s) will help to separate the planar objects with vertical or horizontal direction characteristics. A high k(s) value indicates a planar distribution in horizontal space, such as a flat roof, road, or grass, while a low k(s) value suggests a plane distributed in vertical space like a building façade, and a medium k(s) value suggests classes like gable roofs. The data energy term is defined accordingly:

(10) $$E_{k}(l_{s})=\left\{ \begin{array}{l} \min(\frac{k(s)}{\sigma_{k}},1),\;if\;l_{s}\;belongs\;to\;vertical\;plane\;classes\\ \min(\frac{1-k(s)}{\sigma_{k}},1),\;if\;l_{s}\;belongs\;to\;horizontal\;plane\;classes\\ 0,\;otherwise \end{array}\right.$$

where $\sigma_{k}$ is the empirically determined normalization factor.

According to the nature of the urban environment and LiDAR data characteristics, objects from classes such as road, grass, and building roofs are usually the lowest visible surfaces in a vertical sequence, while objects from the tree class always have ground or roof objects underneath them. A lowest visible object index v(s) is derived from the topology relations among these classes. The v(s) is an indicator function, which gets value 1 only the object *s* belongs to the ground classes. Another data energy $E_{r}(l_{s})$ term can be described by

(11) $$E_{r}(l_{s})=\left\{ \begin{array}{l} \alpha ,\;if\;l_{s}=\;tree,\;v(s)=1\;\\ \beta ,\;if\;l_{s}=ground\;or\;building\;classes,\;v(s)=0\\ 0,\;otherwise \end{array}\right.$$

All the energy terms described above range from 0 to 1. With the smooth energy term $E_{o}(l_{s},l_{t})$ the top layer energy minimization is formulated as

(12) $$E(L_{2})=\sum_{s\in S}\left(E_{d}(l_{s}\right)+E_{h}(l_{s})+E_{g}(l_{s})+E_{k}(l_{s})+E_{r}(l_{s}))+\sum_{s,t\in N_{S}}w_{st}E_{o}(l_{s}\neq l_{t})$$

where *N_S_* is the neighborhood for objects, and $E_{o}(l_{s},l_{t})$ is defined as an indicator function. $w_{st}$ is inverse distance weight similar to Equation (6), and the distance is measured as the minimum distance between segments.

### 2.5. Classification

Since the classification of the point cloud is based on the two-layered graph structure and achieved by minimizing the energy framework described above, the graph cuts algorithm is employed to label both the top layer graph and the bottom layer graph in this paper. To solve the multiple-labelling problem, the α-expansion algorithm that extends from the popular binary-labelling is adopted [29].

The framework first runs graph cuts on the bottom layer to optimize the initial classification and cluster the points into objects. Based on the spatial constraints that reflect the real urban environment, the graph cuts algorithm is then performed on the top layer to optimize the labelling of the objects. The optimized labelling of the objects is then fed back to individual points; thus, the bottom layer graph is modified accordingly. Iterative graph cuts around the top layer graph and bottom layer graph are performed until the classification of the points meets the minimum energy requirements.

Reconstruction of the bottom layer graph during the iteration process is a key step in the proposed method. Since the optimization process of the top layer will change the labelling of some objects, the bottom layer requires the graph structure to be modified to indicate these changes. To efficiently use the feedback from the top layer and simplify the data process, only the data cost term of the points is considered and modified in the bottom layer graph. If the labelling of an object *s* changes from $l_{s}$ to a different class ${l_{s}}^{\prime }$, the data cost term of a point p∈s in the bottom layer will change accordingly. We let $D_{p}{\left(l_{p}\right)}^{\prime }$ denote the modified point data cost, and the reformation of bottom layer graph structure is defined as

(13) $$D_{p}{\left(l_{p}\right)}^{\prime }=\left\{ \begin{array}{l} D_{p}(l_{p})+\theta ,\;l_{p}=l_{s}and{l_{s}}^{\prime }\neq l_{s}\\ D_{p}(l_{p})-\theta ,\;l_{p}={l_{s}}^{\prime }and{l_{s}}^{\prime }\neq l_{s}\\ D_{p}(l_{p}),\;otherwise \end{array}\right.$$

where θ is an adjustment parameter. This formula helps to increase the data cost of original class $l_{s}$, thus decreasing the opportunity of points in the object being re-labelled to class $l_{s}$ again. At the same time, the formula helps to decrease the data cost of the new class ${l_{s}}^{\prime }$ to make the points in s more likely to be labelled as ${l_{s}}^{\prime }$.

**Algorithm 1.** Iterative process of two-layered graph cuts optimizationInput: Point data set P with initial labelling $L^{0}$.Input: Point neighborhood system $N_{P}$, and object neighborhood system $N_{S}$.Input: Normalization factors for object-based features: $\sigma_{hm}$, $\sigma_{ht}$, $\sigma_{hg}$, $\sigma_{g}$, and $\sigma_{k}$.Output: Optimal labelling L for P. Build the bottom layer graph from the initial labelling $L^{0}$ and neighborhood system $N_{P}$, run graph cuts by energy minimizing (Equation (4)), tag all points as ungrouping, and set t=0.Cluster points with ungrouping tag to objects according to point labelling $L^{t}$, normal, and a cluster tolerance; add new objects to the top layer graph.Calculate features and set the energy terms (Equation (7)~(11)) for the new added objects.Build the top layer graph from the object energy terms and object neighborhood system $N_{S}$ and run graph cuts to optimize the labelling of objects by energy minimization (Equation (12)).For each object s, check the label before ($l_{s}$) and after ($l_{s}^{\prime }$) running the top layer graph cuts. If the object label has changed (1) update the data cost of the point in s (Equation (13)), (2) tag points in s and its neighborhood as an ungrouping, and (3) remove s and its neighborhood from the upper layer graph.Set t=t+1 and build a sub-graph using ungrouping points at the bottom layer, run graph cuts for sub-graph to optimize the labelling, and update the labelling of points data $L^{t}$.If $L^{t}!=L^{t-1}$, go back to Step 2.Set $L=L^{t}$.

Besides the modification of the energy terms, determining the subset of points that need to be optimized is another issue for bottom layer reconstruction. Rebuilding the whole bottom layer graph is time costly and unnecessary. An adaptive local modification approach is adopted in the workflow. The proposed method only considers objects with changed labels during the reconstruction of the bottom layer graph. A sub-graph, which is part of the main graph at the bottom layer, is built for points in label-changed objects and related neighborhood objects. Then, the graph cuts algorithm is performed on the sub-graph to optimize the labelling of these specific points. The optimized sub-graph is then merged to the main graph; thus, the whole bottom layer is updated. The iterative process of the bottom and top layer graph cuts is illustrated in Algorithm 1.

## 3. Data and Experiments

### 3.1. Data Sets

**Figure 2:**
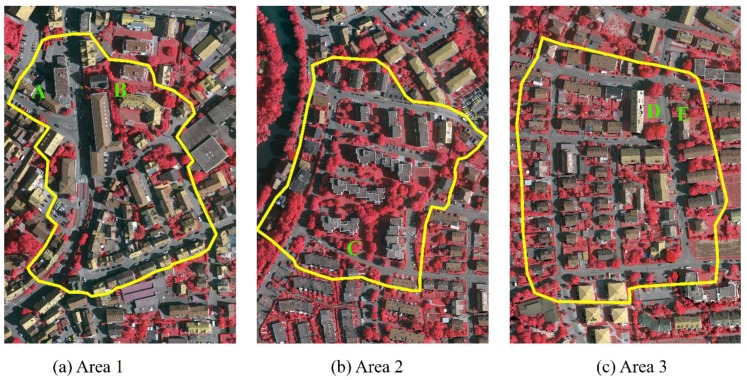
The three test sites in Vaihingen: inner city (**a**), high rise (**b**), and residential (**c**).

The proposed two-layered graph cuts framework (TLGC) was evaluated on benchmark test data sets provided by the ISPRS Test Project on Urban Classification and 3D Building Reconstruction [40]. Three sites over Vaihingen, Germany with different scenes were selected to evaluate the detail performance of the proposed method (Figure 2). Area 1 was characterized by dense buildings with complex shapes. Area 2 showed a few high-rising residential buildings surrounded by trees. Area 3 was a residential area consisting of small, detached houses. Area 1, Area 2, and Area 3 consisted of 246,390, 362,106, and 461,699 points, respectively. Locations (A–E) represent instances that are further inspected in Section 4.3.

Besides the three Vaihingen sites, the entire Toronto data set was used to validate the performance of the proposed method for large-scale urban point cloud. The entire Toronto data set consisted of over 8 million points and covered an area about 1.45 km^2^. Two sites located within the Toronto data set were also selected to further inspect the performance of our method for different types of urban regions. Area 4 contained a mixture of low and high story buildings, showing various degrees of shape complexity. Area 5 was a typical example of a cluster of high-rise buildings in a modern mega city. Area 4 and Area 5 consisted of 1,408,628 and 1,342,622 points, respectively.

In all five test sites, benchmark reference data were only generated for 2D objects. To evaluate the 3D LiDAR data classification results, the point cloud was labelled manually in ArcGIS software. Considering the possible categories of the objects existing in the three test sites, eight object classes were discerned: gable roof, flat roof, building façade, trees, low vegetation, grassland, roads, and cars. The low vegetation also included fences around yards.

### 3.2. Experiments

In the experiments, the Joint-Boost classifier needed to be trained for the purpose of the initial classification of the point cloud. Since the Joint-Boost classifier is a point-based approach, 10% of the points from each class were randomly selected to train the classifier, and the rest of the points were employed for accuracy assessment. Due to the variations of the point density and the urban scenario, it was inappropriate to adopt a unified neighborhood system on the different data sets. An essential parameter, namely the neighborhood size, needed to be evaluated in the experiments. Thus a proper neighborhood would be selected to efficiently capture the local features and context information of the experimental data. Considering the nature of the experimental data, neighborhood sizes between 0.6 and 3.1 m with a step of 0.5 m were chosen in the experiment; thus, six neighborhood sizes were compared.

At the top layer, most of the energy terms except $E_{d}(l_{s})$ were defined according to the inherent constraints of the urban environment. These energy terms employ normalization factors for the calculation of energy values. For the height term $E_{h}(l_{s})$, $\sigma_{ht}$, $\sigma_{hm}$, and $\sigma_{hg}$ were employed to discriminate the objects from different height level. The top level objects such as trees or roofs always have height (h(s)) over 3 m, the middle level objects such as cars or low vegetation have height ranging [0.5 m, 2.0 m], and the ground level objects such as roads or grass have height approaching 0. Fuzzy theory was employed to calculate the height term $E_{h}(l_{s})$, thus our algorithm was insensitive to these three height factors. Empirically, $\sigma_{ht}$, $\sigma_{hm}$, and $\sigma_{hg}$ set as 3, 1.5, and 0.2 m, respectively. The objects with planer surfaces, such as roofs, roads or grass, have planarity (g(s)) approaching 0. In the meantime, some planar objects showing horizontal distribution, such as a flat roofs, roads, or grass have direction (k(s)) approaching 1, while some planar objects showing vertical distribution, such as building façade, have k(s) approaching 0. Similarly, fuzzy theory was introduced to calculate the energy terms $E_{g}(l_{s})$ and $E_{k}(l_{s})$. The optimal values for $\sigma_{g}$ of $E_{g}(l_{s})$ and $\sigma_{k}$ of $E_{k}(l_{s})$ were both set as 0.1. The lowest visible index (v(s)) indicates whether an object is lowest visible in vertical sequence. Thus, α, β of $E_{r}(l_{s})$ were both determined to be 1.

The proposed framework was actually composed of three parts, including the initial classification using the Joint-Boost algorithm, contextual optimization at the bottom layer, and object level optimization at the top layer. To address the advantages of the proposed combined architectures, an “ablation study” introduced by Li et al. [41] was used to compare the classification results from different phases. These three phases included the initial classification by the Joint-Boost algorithm (Phase I), the first round of the bottom layer optimization described as Step 1 in Algorithm 1 (Phase II), and the last round of top layer optimization (Phase III). Actually, Phase I implemented a pure point-based classifier, Phase II optimized the point-based classification with contextual information, and Phase III fully implemented the proposed TLGC method. Besides the combined architecture, the iterative optimization process around the top layer and bottom layer was also evaluated. The classification accuracies were calculated for each round of iteration to address the efficiency of optimization. The experiments were performed on a computer with an Intel core i5, 2.8 GHz CPU, and 16 GB RAM.

## 4. Results and Discussion

### 4.1. Performance of the Combined Architecture

In this section, the classification results from three phases of the proposed framework are compared to evaluate the contributions of the combined architecture. The three study areas in Vaihingen were classified independently with a neighborhood size of 1.2 m in Phase II and Phase III, and the overall accuracies (OA) as well as the Kappa accuracies [42] can be seen in Table 2. The results show that the combination optimization obviously improved the classification accuracies at all three test sites. The overall accuracy and Kappa statistic both agree with these improvements. The accuracies achieved significant increases from Phase I to Phase II, as well as from Phase II to Phase III. Phase III achieved an overall accuracy of 82.1%, 80.8%, and 82.4% for Areas 1, 2, and 3, respectively. The OA differences between Phase III and Phase II at the three test sites were 2.0%, 2.8%, and 2.6% respectively, while the differences between Phase II and Phase I are 3.4%, 2.0% and 2.9%, respectively. The Phase III Kappa statistics were 76.7%, 76.2%, and 78.2% for Areas 1, 2, and 3, respectively. The differences in Kappa statistics between Phase III and Phase II were 2.7%, 3.2%, and 3.1%, respectively, for the three test sites, while the differences between Phase II and Phase Iwere 4.3%, 2.4%, and 3.4%, respectively. The variations in the Kappa statistics were similar to the overall accuracies. However, the increase in Kappa statistics was slightly higher than the increase in overall accuracies.

The completeness and correctness accuracies [42] calculated for the combined Area 1, Area 2 and Area3 are shown in Table 3. The best values are highlighted in bold. The experiments show that Phase II improved the accuracies for all classes compared with Phase I. Phase I is a typical point-based classifier, whereas Phase II combines the contextual information for optimization. This shows that the contextual information conveyed by the neighborhood points can efficiently handle the sparse and noisy point clouds, giving spatial continuity. Phase III improved the accuracies in most classes, except for the completeness of the facade (−0.5%), cars (−3.3%), and road (−0.1%), and the correctness of grassland (−0.2%) and road (−0.1%) were slightly lower. In particular, building roofs (including flat and gable roofs), trees, and low vegetation benefitted from the object level optimization in Phase III.

The top layer optimization introduced object features, including heights, planarity, and vertical sequences, to improve the classification of specific classes. The heights helped to improve the discrimination between trees and low vegetation despite their similar intensity and texture characteristics. Combined with planarity, the discrimination between trees and roofs was enhanced. Although the completeness of the facade slightly decreased, the correctness increased obviously. This indicates that the planarity indices also benefitted the facade class. Since no object features were extracted to help separate the ground-based classes, including grassland and road, the iterative optimization process smoothed the boundaries of these classes and small patches disappeared. Thus, the accuracies of the grassland and road showed no improvements and even decreased slightly in Phase III.

### 4.2. Performance of Iterative Optimization by Local Modification

**Figure 3:**
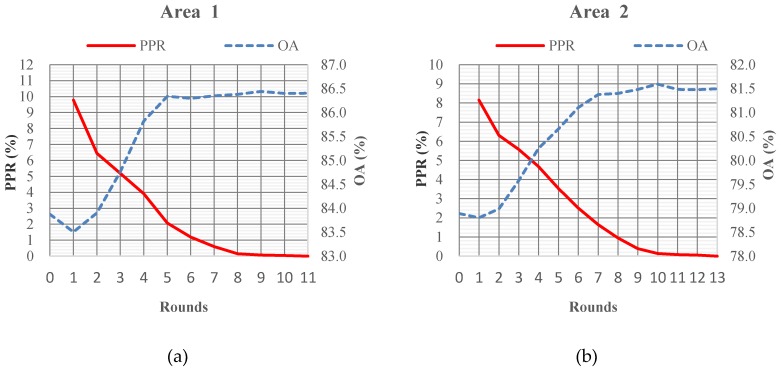

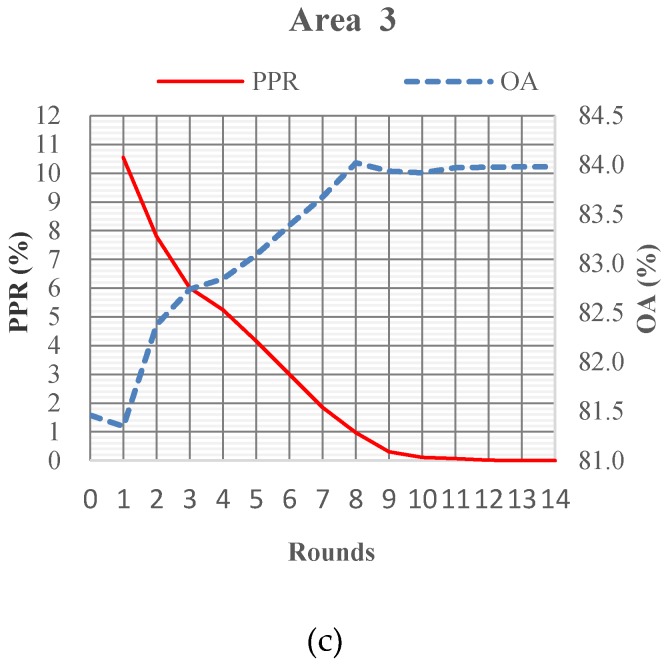
Performance of iterative optimization around the top layer and bottom layer in three Vaihingen sites: (**a**) Area 1, (**b**) Area2 and (**c**) Area 3. PPR(the percentages of points involved in reclassification) and OA( the overall accuracy)

The proposed TLGC method employs iterative optimization to improve the classification accuracy. Each round of iteration includes top layer graph cuts and bottom layer graph cuts. The performance of the iterative process with a neighborhood size 2.0 m tested on Vaihingen data sets is shown in Figure 3. The overall accuracy (OA) for the three study areas slightly decreased in the first round and then quickly reached the maximum value. The maximum accuracy was achieved in rounds 9, 10, and 8, respectively, for the three study areas. After the first round, the percentages of points involved in reclassification (PPR) at the bottom layer were all under 11%–9.8%, 8.2% and 10.6%, respectively, for the three study areas. Then, the number of points requiring reclassification decreased rapidly. After three rounds, the PPR values for the three study areas were 5.2%, 5.57%, and 6.0%, respectively. The PPR values for the three areas were all under 1% after eight rounds of optimization.

The slight decrease in the overall accuracy at the first round was caused by the initialization of the objects. The objects were initialized by clustering points according to the labelling, normal, and distance tolerance, as described in Algorithm 1. In some cases, clustering introduced errors. For example, the normal features confuse the clustering of points on the edge of a roof object. Points from different roof objects may be clustered into one mixed object as they meet the labelling, normal, and distance requirements. Besides the roof, other classes were also affected by the initial clustering process to some degree. The iterative process helped to reduce the effect of clustering in several rounds.

The overall accuracy increased by over 2.5% through the iterative optimization process; however, this process did not introduce a huge amount of extra computation. The time performance of the proposed method is shown in Table 4. The time cost of Phase I was less than 0.1 min for all three study areas. While the time cost of Phase II was 6.5, 9.0, and 10.9, min, respectively, compared with Phase II, Phase III only required half the amount of time for the three areas—3.7, 4.2, and 6.0 min, respectively. At the top layer, the graph nodes represent objects, and there were two orders of magnitude difference between the number of nodes and the original data points. At the bottom layer, benefitting from the adaptive local modification in the workflow, a very small percentage of points, less than 11% in the first round, 6% after three rounds, and 1% after eight rounds, was involved in building a sub-graph for optimization. It is worth introducing this computation cost to give a significant improvement in the classification accuracy.

### 4.3. Impact of the Neighborhood Size

In order to reveal the impacts of neighborhood size on the proposed approach, different neighborhood sizes were tested on the three study areas in Vaihingen. The results are shown in Table 5. All three study areas showed similar accuracy variation. The accuracy first increased and then decreased as the neighborhood size increased. The best overall accuracies (86.4%, 81.5%, and 84.0% for Areas 1, 2, and 3, respectively) and Kappa accuracies (82.1%, 77.1%, and 80.1% for Areas 1, 2, and 3, respectively) all occurred at a neighborhood size of 2.1 m, which is about four times the average spacing of the points.

Different neighborhood sizes led to notable variations in classification accuracies. The maximum difference in overall accuracy for the three study areas was 7.7%, 3.6%, and 5.2%, respectively, and the maximum difference in Kappa accuracy was 9.8%, 4.5%, and 6.6%, respectively. The variation in Kappa accuracy was slightly higher than the overall accuracy. There were classes with small object sizes, such as cars, facades, and low vegetation, which were more sensitive to the neighborhood size, resulting in a higher variation in accuracy at the class level. However, these classes only accounted for a small portion of the study area. This explains the difference in variation between the overall accuracy and Kappa accuracy.

**Figure 4:**
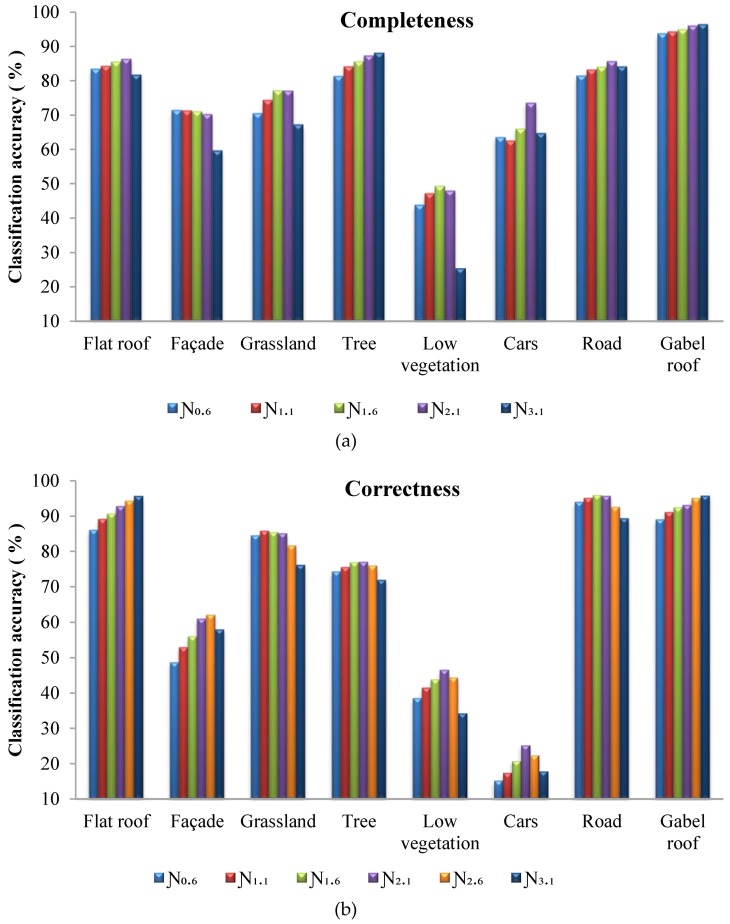
Completeness (**a**) and correctness (**b**) for all classes with different neighborhood sizes in combined Vaihingen sites.

The completeness and correctness for all classes with different neighborhood sizes are shown in Figure 4. Except for the completeness of facades and trees and the correctness of roofs (including the gable and flat roof), the class level accuracies showed similar variation to the overall accuracy, which first increased and then decreased as the neighborhood size increased. The facade, low vegetation, and car showed higher variation than the other classes. This indicates that these classes are more sensitive to the neighborhood size. As the neighborhood size increased, the accuracy of the roof and tree classes was more stable. The correctness of the roof and completeness of trees even showed continuous increases. In contrast to the roofs and trees, the accuracy of grassland and roads decreased when the neighborhood size was larger than 2.1 m.

There are two reasons for the neighborhood-size-related variation in the accuracy. The first is the smoothing function that was put into effect by the graph cuts algorithm. Both the bottom layer and top layer of the proposed method introduced the graph cuts algorithm for energy minimization. As the neighborhood size increased, the smoothing of the classification was enhanced. Over-smoothing sieves small or narrow objects and blurs the boundaries between large objects. The other reason was that the energy terms derived from object features intend to preserve the objects according to the natures of different classes. Trees, roofs, roads, and grasslands are classes with large object sizes; however, their responses to the neighborhood size were found to be different. The top layer graph employed planarity indices to separate roofs and trees, while no features were provided to discriminate the roads and grasslands. The second factor explains the accuracy difference in these classes.

### 4.4. Classification Results of the Vaihingen Sites

**Figure 5:**
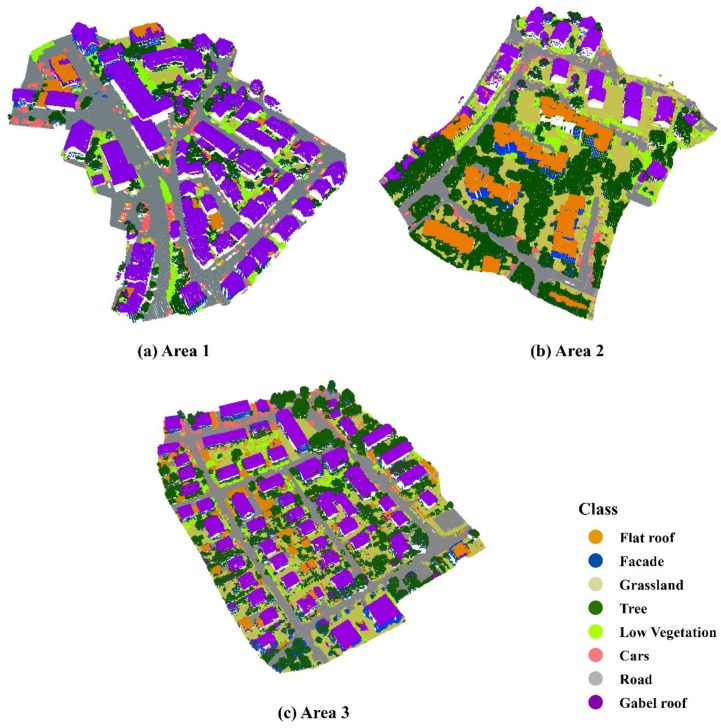
Three-dimensional view of the classification results for the three Vaihingen sites.

The classification results of the three study areas in Vaihingen are depicted in Figure 5. With the best neighborhood size, 2.1 m, the proposed TLGC approach achieved an overall accuracy of 86.4%, 81.5%, and 84.0% for areas 1, 2, and 3, respectively. The Kappa indices were 82.1%, 77.1%, and 80.1%, respectively. Misclassification was first observed between the roofs (including gable roofs and flat roofs) and trees. At the ground level, confusion between roads and grasslands was detected. The incorrect assignment of low vegetation to cars was also significant.

**Figure 6:**
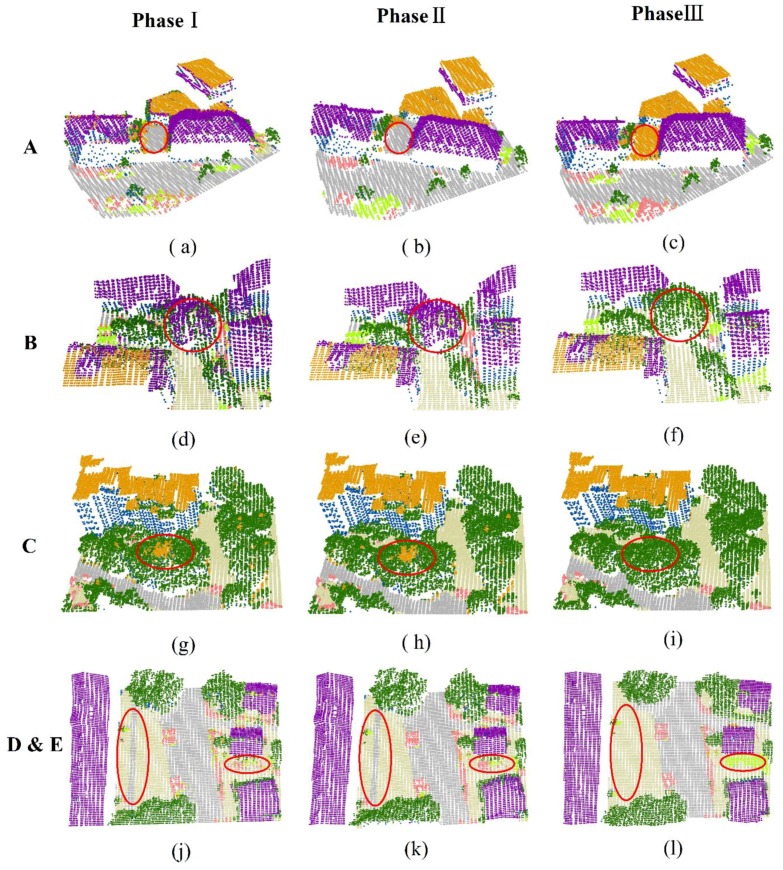
Comparisons of some classification results for the three phases. (**A**–**E**) correspond to the locations marked in Figure 2.

Some classification instances were compared among the three phases, as described in Section 4.1. The results are shown in Figure 6. The selected instances included connected buildings with different heights (A), building roofs covered with tree-tops (B, C), grasslands with narrow roads (D), and low vegetation (E). According to the results from Phase I to Phase III, TLGC led to improvements in most instances. However, it also introduced errors in some instances.

Connected buildings are usually composed of gable and flat roofs. The heights of these roofs showed obvious variation. Complex conditions affected the efficient classification of connected buildings. There was a small building with a flat roof between two gable roof buildings in (A). Some of the points of the flat roof were classified as roads in Phase I, and the contextual optimization enhanced the misclassification by labelling the whole roof as a road in Phase II. Top layer optimization corrected this misclassification with the help of the object energy terms (e.g., $E_{r}(l_{s})$) in Phase III. According to common sense, roads should be a ground class, while roofs should be a non-ground class.

The misclassification between tree and building elements (roofs, facades) was common in the three study areas. In Phase I, point features were used to classify the point cloud. Most of these features were defined with sphere or cylinder neighborhoods. Deviation from a local plane is a key feature to discriminate the roof class from the tree class. However, surfaces with deviations smaller than a roof can also be derived from canopies of big trees. This challenges the classification of roof classes and trees. In (B, C), a great number of points from tree canopies were classified as roof points in Phase I. Contextual optimization at the bottom layer rectified the labelling of some of small segments from roofs to trees in Phase II; however, large segments remained incorrectly labelled. In contrast, the object-based optimization at the top layer rectified most of the misclassification between roofs and trees in Phase III, including small segments and large segments. In Phase III, the lowest visible indicator was introduced as a feature in the top layer to solve this problem. Since there are always segments under the tree canopies, the misclassified tree canopies can be easily rectified by energy terms (e.g., *E_r_*(*l_s_*)). However, the lowest visible indicator feature also has limits. In (C), some facades protruded from the buildings and were incorrectly classified as trees in Phases I and II. Since there were lower segments under these bulges, they remained incorrectly classified In Phase III.

The confusion between low vegetation and cars was also significant after optimization. The accuracy of these two classes, as shown in Table 3, was the lowest in the study areas. In particular, the correctness accuracy for the cars was under 20%–13.6%, 16.0% and 18.0% in the three phases, respectively. There were low vegetation objects (including fences and bushes) around a house in (E). Most of the low vegetation objects were classified as cars in Phases I and II, and only some of these objects were rectified in Phase III. Low vegetation objects with rough surfaces like shrubs were well discriminated by the object features in the top layer graph. However, the fences were difficult to discriminate from the cars, because they also have smooth surfaces. Therefore, the bushes were rectified and the fences remained incorrectly classified in Phase III. Fences are common in residential areas; thus, significant confusion was observed in the three study areas.

There was a narrow road crossing the grassland in (D). It was correctly classified in Phases I and II. However, this road disappeared in the final results after iterative optimization. The LiDAR data intensity was the most important point feature for discriminating between road and grassland. Since the intensity is sensitive to the incidence angle, it confused these two classes. Moreover, the iterative graph cuts process in Phase III introduces a smoothing effect into the results. It blurred the boundary between the road and grassland, and the narrow road disappeared in the final results.

According to the results from Phase I to Phase III, TLGC improved the classification in most instances. The complex urban environment and limitation of the point-based features resulted in some obvious misclassifications in Phases I and II. In Phase III, the spatial constraints derived from the real urban environment, such as the height, planarity, and topology relation, showed the ability to reduce these misclassifications at the object level. The normalization factors, which were used to calculate the energy terms from the spatial constraints, can be empirically defined and tweaked according to different urban scenes. Compared to the feature learning methods, the empirically defined normalization factors directly use the characteristics of urban object types, thus avoiding mass training samples and a high computational effort.

The performance of the proposed method depended largely on the efficiency of the spatial constraints (object features). Due to the complexity of the urban environment, it was difficult to extract appropriate object features to describe the 3D structure. In this study, four object features, including height, planarity, direction and lowest visible indicator, were employed to optimize the classification in the top layer. However, these object features were incapable of covering the discrimination among all classes, e.g., road and grass. In addition, the iterative process tended to over smooth among these classes. The avoidance of over smoothing for specific classes could be part of our future work.

### 4.5. Validation Results of the Toronto Sites

**Figure 7:**
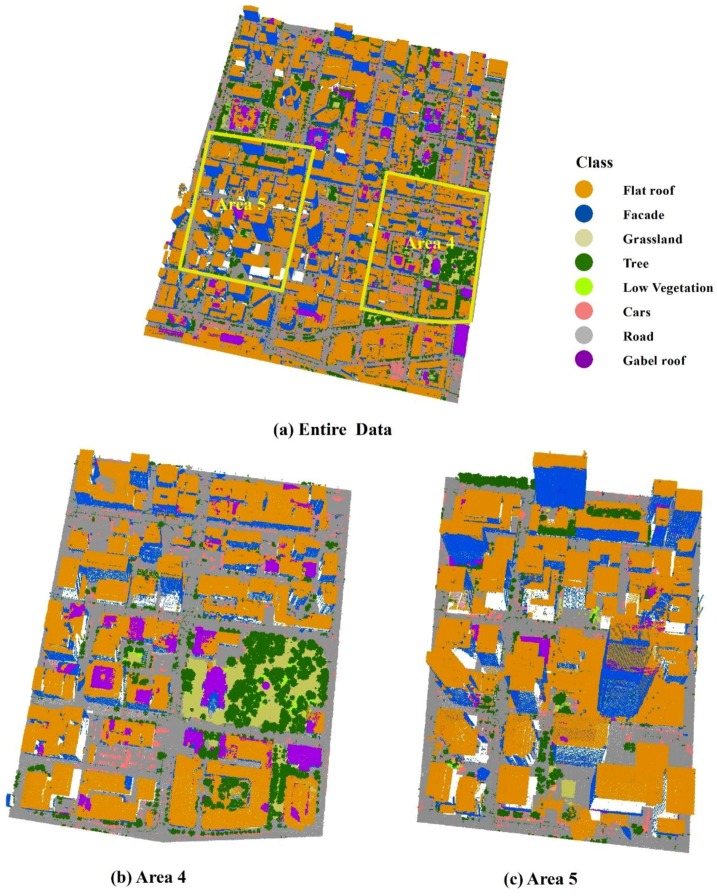
Three-dimensional view of the classification results for the Toronto data set.

The classification results of the entire Toronto data set (Entire Data) and zoomed Area 4 and 5 are depicted in Figure 7. Area 4 and 5 were used to inspect the performance of the proposed method for the different types of urban regions. Misclassification was first observed between the roofs and facades at the rooftop level. Some flat roofs and façades were wrongly classified as gable roofs. The Incorrect assignment of road and grassland was detected in Area 4. Narrow roads crossing the grassland disappeared in the park region, which was similar to Vaihingen sites. At the ground level, the confusion between cars and other small size objects was also significant.

The classification accuracies calculated for the Entire Data, Area 4 and 5 are shown in Table 6. The overall accuracies (88.3%, 89.4%, 91.8% for Entire Data, Area 4 and 5, respectively), as well as Kappa accuracies (83.6%, 86.0%, and 87.9% for Entire Data, Area 4 and 5, respectively), are higher than that of the Vaihingen sites. There are two reasons for the increase of the accuracies. First, the Toronto data set was captured in the central area of City of Toronto. High rising buildings and roads dominate the test data set. The complex vertical overlapping or mixing between the trees and buildings are seldom. Comparing to Vaihingen data set, the misclassification between the tree and building types (façade, flat roof and gable roof), significantly decreased. Second, the low vegetation and grass only cover a small area of the data set. Due to the small proportion of the low vegetation (0.6%, 0.8% and 0.3% of total points in Entire Data, Areas 4 and 5, respectively) and the grass (2.2%, 7% and 0.4% of total points in Entire Data, Areas 4 and 5, respectively), incorrect assignment of low vegetation to cars and trees, as well as incorrect assignment of grassland to road, were obviously less than that of the Vaihingen data set. The gable roof was the only class that obtained much lower accuracies. It was caused by the wide variety of rooftop structures in this region.

The computation time on the Entire Data for Phases I, II and III were 2.0, 108.3 and 35.7 min, respectively. Large-scale point cloud introduced huge computation for the proposed method. In Phases II, it took over 100 min to perform the graph formulation and the graph cuts optimization on the Entire Data that consisted of over 8 million points. In future work, techniques such as block processing and parallel computing will be employed to improve the computation efficiency and make the proposed method more appropriate for the large-scale point cloud.

### 4.6. Comparison of Accuracy with Other Methods

The comparison of the proposed method and other state-of-the-art methods was performed on the ISPRS benchmark dataset. Since the classification schema of our work was different from the one used in the labelling benchmark dataset, they were unified to make the comparison possible. The gable roofs and flat roofs in our work were merged into the category roofs. The fence-hedges and shrubs in the benchmark data were merged into the category low vegetation. The low vegetation and impervious surfaces in the benchmark data referred to grassland and roads, respectively. The proposed method were compared with five state-of-the-art methods, including UM, LUH, NANJ2, WhuY3, and BIJ_W. The UM method employs supervised machine learning for point cloud classification, and the features are extracted from point-attributes, textural analysis, and geometric attributes [43]. The LUH method [26] uses a hierarchical framework based on conditional random fields and integrates object features by voxel cloud connectivity segmentation [44] for classification. WhuY3 [45], NANJ2 [19], and BIJ_W [20] are deep learning technology based methods. WhuY3 and NANJ2 both generate 2D images from point cloud features; thus, convolutional neural networks run on feature images for classification. The main difference between these two deep learning methods is that NANJ2 employs a multi-scale convolutional neural network to learn features at various scales. BIJ_W uses a deep learning process similar to that of PointNet [46] that avoids rasterizing the irregular points. A novel spatial pooling is employed to capture multi-scale features for classification.

The results from the proposed method and other state-of-the-art methods are shown in Table 7. The highest values and second highest values are highlighted in bold, except NANJ2. The NANJ2 method obviously outperformed the other methods; however, it combines extra RGB features obtained from the corresponding orthoimages for classification purposes. Since spectral features will improve the discernibility of some classes, it is not suitable to simply consider the accuracy of NANJ2. Besides NANJ2, the proposed TLGC method achieved an overall accuracy of 83.2%, which was better than that of the rest of the methods. For the F1-scores, the TLGC method obtained the highest and second highest values, except for low vegetation, cars, and road. The LUH method also performed well in most of the classes with an overall accuracy of 82.7%. WhuY3 and BIJ_W, which are recognized as deep learning methods, achieved high overall accuracy, 82.9% and 82.4%, respectively. However, these two methods did not demonstrate advantages for F1-scores compared with TLGC and LUH.

Actually, LUH and TLGC are both point-based and object-based hybrid methods based on hierarchical structures. The difference is that LUH is a purely data-driven approach using Conditional Random Fields, while TLGC uses the nature of urban classes to empirically define spatial constraints (e.g., shape, height, and topology) to conduct the classification of point clouds at the object level. TLGC integrates the merits of the data-driven and knowledge-driven approaches to make the classification results more reasonable. Therefore, classes can be well discerned by the spatial constraints, achieving remarkable improvements in the classification accuracy (e.g., roofs, facades). The deep learning technology based methods usually convert the raw points into 2D images or regular volumetric representation (e.g., WhuY3, NANJ2) to conduct the convolution operations. The conversion process results in the loss of crucial and discriminative geometric details. Recently, some deep neural networks are designed to directly manipulate raw point cloud data (e.g., BIJ_W). However, such kind of methods largely treat points independently or inefficiently capture local features. Comparing with the point-based and object-based hybrid methods (TLGC, LUH), the deep learning technology based methods do not show their superiorities. This also indicates that object-based evaluation is essential for point cloud classification.

## 5. Conclusions

In this paper, a two-layered hierarchical framework (TLGC) was proposed to classify point clouds in the urban environment. In this framework, at the bottom layer, a graph is built on the original data points, while at the top layer, an object-based graph is built on the clusters of points derived from the bottom layer. The graph cuts algorithm iteratively runs around the top layer and bottom layer to optimize the classification. The proposed TLGC framework has two main merits. First, it has the ability to flexibly combine local contextual information and object-based features to build a hybrid classifier. Second, the adaptive local modification has been proven to be an efficient way to propagate information around a hierarchical framework, and it also help to avoid the need for huge computational effort. These merits make it possible to perform the TLGC method on clouds with mass points. The experiments demonstrate that the proposed method is capable of producing classification results with high accuracy and efficiency. It provides the potential for point cloud classification in complex urban environments.

Currently, several spatial constraints at the object level, including the heights, planarity, and lowest visible indicator are employed as features to optimize the classification at the top layer. However, due to complicated urban environment and nature of the point cloud, these features cannot efficiently discriminate among all classes. For example, grasslands and roads are not well separated by the above features, and the confusion between low vegetation and cars is still significant. Future work will consider incorporating deep learning features at the object level to achieve better classification.

## Figures and Tables

**Table 1:** Synthesis of LiDAR features for initial classification.

Feature Type	Name	Neighbourhood Definition
Intensity features	Intensity	-
	Intensity variance	Sphere
Echo features	Number of echoes	-
	Ratio of echoes	-
Height features	Height above ground	-
	Height variance	Cylinder
	Curvature	Cylinder
Eigenvalue features	Eigenvalues	Sphere
	Omnivariance,	Sphere
	Planarity	Sphere
	Anisotropy	Sphere
	Sphericity	Sphere
Plane features	Normal vector variance	Sphere
	Residuals to the local plane	Sphere
Density features	Point density	Cylinder/sphere
	Point density variance	Cylinder/sphere

**Table 2:** Comparison of the overall accuracies and Kappa statistics for the three phases in three Vaihingen sites.

Phase	Area 1		Area 2		Area 3	
	OA (%)	Kappa (%)	OA (%)	Kappa (%)	OA (%)	Kappa (%)
I	76.7	69.7	76.0	70.6	76.9	71.7
II	80.1	74.0	78.0	73.0	79.8	75.1
III	82.1	76.7	80.8	76.2	82.4	78.2

**Table 3:** Comparison of the classes’ accuracies for the three phases in combined Vaihingen sites. Bold numbers show the highest values among the three phases.

	Phase I	Phase II	Phase III
OA (%)	76.6	79.2	81.6
Kappa (%)	71.5	74.7	77.5
Flat roof (%)	82.9/78.0	84.6/81.7	**85.3/89.5**
Façade (%)	71.5/41.5	**72.8**/45.4	72.3/**53.6**
Grassland (%)	64.3/84.1	69.6/**86.5**	**75.88**/86.3
Tree (%)	77.2/73.0	79.2/74.2	**82.6/76.5**
Low vegetation (%)	40.0/34.9	41.9/37.9	**48.8/43.6**
Cars (%)	67.5/13.6	**68.5**/16.0	65.2/**17.9**
Road (%)	80.5/94.0	**83.2/95.8**	83.1/95.7
Gable roof (%)	93.3/86.2	94.6/88.0	**94.9/90.2**

**Table 4:** Computation time of the proposed method in three Vaihingen sites.

	Phase I (min)	Phase II (min)	Phase III (min)	Total Cost (min)
Area 1	0.1	6.5	3.7	10.3
Area 2	0.1	9.0	4.2	13.3
Area 3	0.1	10.9	6.0	17

**Table 5:** Overall accuracies and Kappa statistics with different neighbourhood size in three Vaihingen sites.

Neighborhood (m)	Area 1		Area 2		Area 3	
	OA (%)	Kappa (%)	OA (%)	Kappa (%)	OA (%)	Kappa (%)
0.6	78.7	72.3	78.3	73.2	80.3	75.6
1.1	81.6	75.8	80.0	75.3	82.3	78.1
1.6	83.6	78.5	81.0	76.5	83.6	79.6
2.1	86.4	82.1	81.5	77.1	84.0	80.1
2.6	86.1	81.6	79.3	74.4	83.4	79.4
3.1	83.9	78.7	77.9	72.6	78.8	73.5

**Table 6:** Validation results of Toronto data set: completeness, correctness, overall accuracy and Kappa.

	Entire Data	Area 4	Area 5
OA (%)	88.3	89.4	91.8
Kappa (%)	83.6	86.0	87.9
Flat roof (%)	90.1/92.4	90.5/94.6	91.0/95.7
Façade (%)	83.3/77.8	84.2/77.7	86.3/79.2
Grassland (%)	81.1/80.7	81.4/82.5	79.8/83.0
Tree (%)	91.0/85.1	90.2/88.8	88.0/87.6
Low vegetation (%)	48.5/56.1	51.6/52.0	48.9/40.7
Cars (%)	58.6/40.5	63.8/52.7	69.3/50.3
Road (%)	93.4/95.2	95.0/94.6	97.2/98.3
Gable roof (%)	80.1/69.2	77.8/73.9	65.7/40.1

**Table 7:** Comparison of TLGC method and other state of art methods on ISPRS benchmark dataset in terms of F1-scores and OA. Bold numbers show the highest values and second highest values among different methods except NANJ2.

	TLGC	UM	LUH	WhuY3	BIJ_W	NANJ2
OA (%)	**83.2**	81.8	82.7	**82.9**	82.4	86.4
Roof (%)	**94.5**	92.0	**94.2**	93.4	92.2	93.6
Façade (%)	**65.3**	52.7	**56.3**	47.5	53.2	42.6
Grassland (%)	**80.9**	79.0	77.5	**81.4**	78.5	88.8
Tree (%)	**81.9**	77.9	**83.1**	78.0	78.4	82.6
Low vegetation (%)	47.2	49.8	**55.0**	46.5	**53.4**	65.9
Cars (%)	37.6	47.7	**73.1**	**63.4**	56.4	66.7
Road (%)	90.4	89.1	**91.1**	90.1	**90.5**	91.2
